# Pt Nanoparticles on Multi-Walled Carbon Nanotubes with High CO Tolerance for Methanol Electrooxidation

**DOI:** 10.3390/molecules29215015

**Published:** 2024-10-23

**Authors:** Pingping Yang, Shiming Dong, You Shu, Xuejiao Wei

**Affiliations:** 1 College of Chemistry and Materials Engineering, Huaihua University, Huaihua 418000, China; yangpingping202211@163.com (P.Y.); dsm20032022@163.com (S.D.); 2 Hutian Middle School, Huaihua 418000, China

**Keywords:** Pt nodes, Pt–O bond, ethylene glycol-based deep eutectic solvents, high CO resistance, direct methanol fuel cells

## Abstract

Anode catalysts are important for direct methanol fuel cells (DMFCs) of energy conversion. Herein, we report a novel strategy by ethylene glycol-based deep eutectic solvents (EG-DESs) for the fabrication of a multi-walled carbon nanotubes (MWCNTs)-supported Pt nanoparticles catalyst (referred to as Pt/CNTs-EG-DES). The Pt/CNTs-EG-DES catalyst provides an increased electrochemically active surface area (ECSA) and shows remarkably improved electrocatalytic performance towards methanol oxidation reaction compared to Pt/CNTs-W (fabricated in water) and commercial Pt/C catalysts. The improved performance is attributed to the generation of more Pt–O bonds which change the electronic states of the Pt atoms and the special node structure that obtains more active sites for a high CO resistance. This study suggests an effective synthesis strategy for Pt-based electrocatalysts with high performance for DMFC applications.

## 1. Introduction

The energy crisis is one of the most urgent problems to be solved [1,2,3,4,5,6,7,8,9,10]. Direct methanol fuel cells (DMFCs) have attracted great interest due to their advantages, low operating temperature, convenient storage and transportation of liquid fuel, high energy density, and low pollution emissions [11,12,13,14,15,16]. The demand for high catalytic performance, long-term durability, and cheap electrocatalysts has driven the development of DMFCs [17,18,19,20,21]. Notably, the platinum bromine nanoparticles with electron-rich properties from bromine bonded with sp-C in graphdiyne (PtBr NPs/Br-GDY) have been fabricated, which show a high CO resistance to methanol oxidation reaction (MOR) [22], and an L1_2_-Pt_2_RhFe intermetallic catalyst with integrated functional components has been demonstrated, which exhibits exceptional CO tolerance [23]. Platinum/poly(3,4-propylenedioxythiophene)/carbon nanobox (Pt/PProDOT/CNB) composites were synthesized by wrapping CNB around PProDOT films via in situ oxidative polymerization [24]. Compared with the previous DMFCs’ anodic methanol oxidation performance, these new studies show better catalytic performance.

At present, platinum (Pt) is still considered to be the best catalyst, while the toxic effect of CO on the catalyst is an inevitable problem [25,26,27,28,29,30]. The CO produced by the reaction covers the active site of the catalyst and significantly inhibits the catalytic activity [31,32]. Therefore, improving the utilization of Pt is a relatively effective way to solve the CO toxicity problem of the catalyst. Additionally, controlling the morphology of Pt to obtain more defect structures is an effective method to improve the catalytic performance of catalysts [33,34,35,36]. Zhang et al. have constructed a kind of novel spiny PtFePd@PtFe/Pt core@multishell 1D NW catalyst with PtFePd displaying remarkable catalytic properties for MOR [36]. Fan et al. prepared Pt nanoparticles with hollow-opened structures (Pt HOSs) as an efficient electrocatalyst for MOR via a facile template-free solvothermal synthesis [33]. Therefore, controlling the morphology of Pt to obtain more defect structures is indeed beneficial to improving the catalytic performance of catalysts.

Deep eutectic solvents (DESs) are a novel class of solvents consisting of quaternary ammonium salts and hydrogen bond donors (e.g., carboxylic acids, amides, and polyols) that have received a lot of attention in electrocatalysis applications due to their remarkable physicochemical properties, which include high conductivity, thermostability, negligible vapor pressure, and wide electrochemical potential windows [37,38,39,40,41]. In particular, we have reported that the Pt_2_CeO_2_ heterojunction nanocluster (HJNS) on multi-walled carbon nanotubes (MWCNTs) in DESs exhibited superior electrocatalytic activity and stability for ethanol oxidation compared to a commercial Pt black catalyst [42]. Furthermore, ultrafine Pt_1_(CeO_2_)_0.5_-decorated multi-walled carbon nanotube nanoelectrocatalysts (Pt_1_(CeO_2_)_0.5_/MWCNTs-D) that were performed using DESs showed excellent electrocatalytic activity [43]. More recently, Liu et al. reported the DES formed by cobalt salt and urea was used as an all-in-one platform for the construction of two-dimensional (2D) [Co-(NH_3_)_4_CO_3_]Cl nanosheets [44]. These catalysts were prepared in a urea–choline chloride DES, and the post-treatment process of excess DESs is quite complicated. It is a challenge to use a class of DES that is easy to post-process to prepare high-performance catalysts.

In this paper, we report for the first time the chemical reduction synthesis in EG-DESs of highly active Pt nanocatalysts supported on MWCNTs. We successfully obtained Pt nanocrystals with a large number of nodes. In addition, the introduction of EG-DESs has been proven to be beneficial to the formation of a large number of Pt–O bonds, which is conducive to improving the catalytic performance of Pt [43]. These results demonstrate that the use of the EG-DES medium significantly changed the morphology and structure of the catalytic nanoparticles.

## 2. Results and Discussion

### 2.1. XRD Analysis of Samples

**Figure 1:**
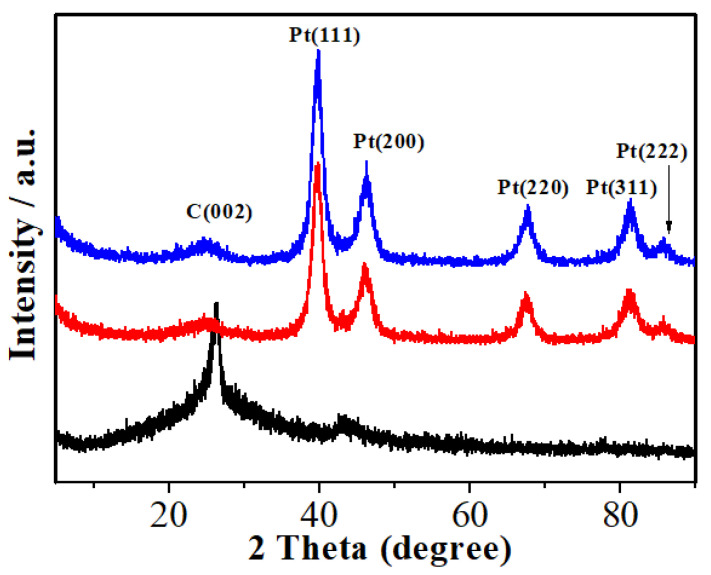
XRD patterns of Pt/CNTs-EG-DES (red), Pt/CNTs-W (blue), and MWCNTsAO (black).

Figure 1 shows the XRD patterns of the Pt/CNTs-EG-DES (red), Pt/CNTs-W (blue), and MWCNTs-AO (black). The peak at approximately 2θ = 26.1° for all samples was due to the C(002) phase [4,43]. Meanwhile, the Pt/CNTs-EG-DES and Pt/CNTs-W show peaks characteristic of Pt(111) at 40.7, (200) at 47.7, (220) at 68.4, and (311) at 82.8, implying that the Pt nanoparticles were successfully supported on the MWCNTs [45]. The average crystallite size of the Pt nanoparticles was determined to be 9.6 for the Pt/CNTs-EG-DES catalyst. In order to better understand MWCNTs, SEM characterization of MWCNTs and Pt/CNTs-EG-DESs was re-performed (Figure S1).

### 2.2. TEM and XPS Characterization of Catalysts

**Figure 2:**
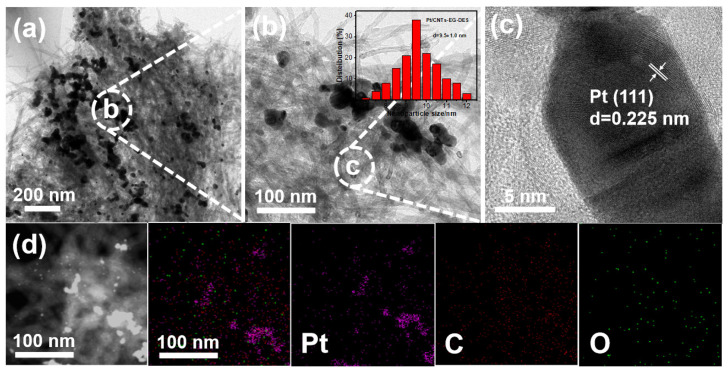
TEM image (**a**,**b**), HRTEM image (**c**), and the corresponding elemental mappings (**d**) of Pt, C, and O of the Pt/CNTs-EG-DES catalyst.

Figure 2 shows the TEM (a, b), HRTEM images (c), and the corresponding elemental mappings (d) of Pt, C, and O of the Pt/CNTs-EG-DES catalyst. As shown in Figure 2a,b, most of the Pt nanoparticles are evenly dispersed on the MWCNTs. The average particle size of the Pt/CNTs-EG-DES catalyst is approximately 9.6 nm, which is very close to the XRD data. The HRTEM image of the Pt/CNTs-EG-DES (Figure 2c) shows the oriented and ordered fringe pattern; the lattice spacing value of 0.225 nm is consistent with that of the Pt (111) planes [43], which further provides proof for the existence of Pt nanoparticles in the Pt/CNTs-EG-DES. These results are supported by HAADF-STEM, where the corresponding elemental mapping images of O, Pt, and C are well displayed in Figure 2d. Furthermore, as shown in Figure S2, for the Pt/CNTs-W catalyst, a large number of Pt nanoparticles are agglomerated together, and some of them are amorphous. Interestingly, the Pt/CNTs-EG-DES was fabricated in the EG-DES, which acts as a surfactant to induce a cluster distribution of Pt nanoparticles. These Pt nanoparticles are connected in a network one after another, and many nodes (Figure S3) and voids are generated in the middle. This network structure is conducive to the rapid flow of electrolytes, which is the main reason for the improvement in catalytic performance [40,43,46,47].

**Figure 3:**
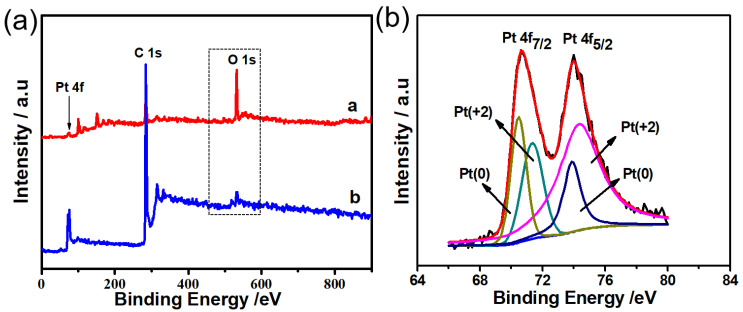
(**a**) XPS survey spectrum of Pt/CNTs-EG-DES (red) and Pt/CNTs-W (blue); (**b**) Pt (4f) (Pt^+2^: Baby blue and pink; Pt^0^: Dark green and blue) spectrum of Pt/CNTs-EG-DES.

XPS measurements were employed to characterize the surface composition and chemical oxidation states of the catalysts. Figure 3a shows the XPS survey spectra of the Pt/CNTs-EG-DES and Pt/CNTs-W catalysts. The signals corresponding to C 1s (283.7 eV), O 1s (531.8 eV), and Pt 4f (73.3 eV) were observed for two catalysts [43,48]. It can be seen that the strength of O in the Pt/ CNT-EG-DES is higher, which indirectly indicates that the proportion of O in the Pt/CNT-EG-DES is higher than that in the Pt/CNT-W. Figure 3b and Figure S4 show the Pt (4f) spectrum for the Pt/CNTs-EG-DES and Pt/CNTs-W catalysts, respectively. The two pairs of peaks indicate the existence of two different Pt oxidation states on the surface. For the Pt/CNTs-EG-DES, the two intense peaks located at binding energies of 70.4 eV (Pt 4f 7/2) and 74.2 eV (Pt 4f 5/2) originated from metallic Pt(0), and the peaks located at 71.5 eV (Pt 4f 7/2) and 74.5 eV (Pt 4f 5/2) were assigned to the Pt(+2) state in the form of PtO or Pt(OH)_2_ [49,50]. Based on the relative areas of these peaks, the percentages of the Pt(0) and Pt(+2) species were calculated to be 38.7% and 61.3%. For the Pt/CNT-W catalyst, the Pt 4f 7/2 peak located at 71.1 eV and Pt 4f 5/2 peak located at 74.6 eV correspond to metallic Pt(0), and the Pt 4f 7/2 peak located at 71.7 eV and Pt 4f 5/2 peak located at 75.5 eV correspond to Pt(+2). The percentages of Pt(0) and Pt(+2) in the Pt/CNTs-W were 59.3% and 40.7%. Evidently, the percentage of Pt(+2) for the Pt/CNT-EG-DES (61.3%) is higher than the percentage of the Pt/CNTs-W (40.7%), indicating that the “specific” condition of the EG-DES favors higher amounts of Pt(+2); this can be supported by the result of the Raman characterization on the Pt/CNT-EG-DES, where a typical Pt–O bond peak (585 cm^−1^) was seen (Figure S4), probably suggesting stronger electron interactions between the Pt nanoparticles and the O [51]. These electron interactions between Pt and O will significantly change the electronic states of the Pt atoms and enhance the electrocatalytic performance of the Pt/CNT-EG-DES for methanol oxidation, and the effect of O will markedly minimize the CO [43].

### 2.3. Electrochemical Characterizations

**Figure 4:**
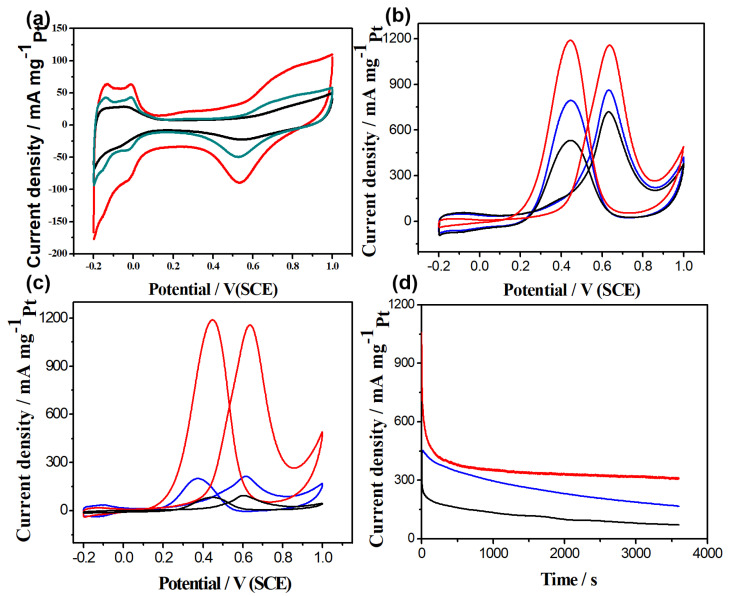
(**a**) Cyclic voltammograms of Pt/CNTs-EG-DES (red), Pt/CNTs-W (blue), and Pt/C (black) catalysts in 0.5 M H_2_SO_4_ solution; (**b**) cyclic voltammograms of Pt/CNTs-EG-DES (red), Pt/CNTs-EG-DES-90 (blue), and Pt/CNTs-EG-DES-25 (black) in 0.5 M H_2_SO_4_ + 0.5 M CH_3_OH solution; (**c**) cyclic voltammograms of Pt/CNTs-EG-DES (red), Pt/CNTs-W (blue), and Pt/C (black) catalysts in 0.5 M H_2_SO_4_ + 0.5 M CH_3_OH solution; (**d**) current–time curves of Pt/CNTs-EG-DES (red), Pt/CNTs-W (blue), and Pt/C (black) catalysts in 0.5 M H_2_SO_4_ + 0.5 M CH_3_OH solution.

Figure 4a shows the cyclic voltammograms of the Pt/CNTs-EG-DES (red), Pt/CNTs-W (blue), and Pt/C (black) catalysts in a N_2_-saturated 0.5 M H_2_SO_4_ solution at a scan rate of 50 mVs^−1^. The typical CV characteristics of Pt were observed with hydrogen adsorption/desorption regions between −0.2 and 0.1 V and Pt oxidation/reduction peaks. The electrochemical active surface area (ECSA) of Pt-based catalysts is calculated by measuring the hydrogen adsorption/desorption charges after double-layer correction [37,52]. Therefore, the ECSA of the Pt/CNTs-EG-DES was calculated to be 52.16 m^2^g^−1^, which is higher than the Pt/CNTs-W (39.32 m^2^g^−1^) and Pt/C (18.3 m^2^g^−1^) catalysts. The larger ECSA of the Pt/CNTs-EG-DES is most likely due to the special Pt node structure on the MWCNTs, which is favorable for MOR [42,45,53,54]. Figure 4b shows the CV curves for MOR on the Pt/CNTs-EG-DES, Pt/CNTs-EG-DES-90, and Pt/CNTs-EG-DES-25 catalysts. The electrooxidation of methanol on these catalysts was characterized by two irreversible current peaks that are similar to those previously reported [43]. The anodic peak in the forward scan at approximately 0.70 V originated from methanol oxidation, and the other anodic peak in the reverse scan at approximately 0.45 V was associated with the oxidation of intermediate carbonaceous species formed during the forward scan. For the Pt/CNTs-EG-DES, the peak current densities of methanol oxidation in the forward and reverses scans are 1101.2 and 1027.5 mA mg^−1^_Pt_, respectively, which are higher than those on the Pt/CNTs-EG-DES-90 (850.1 and 802.7 mA mg^−1^_Pt_) and Pt/CNTs-EG-DES-25 (702.9 and 452.6 mA mg^−1^_Pt_) catalysts. Figure S4 shows the cyclic voltammograms of the Pt/CNTs-EG-DES (red, ethylene glycol/choline chloride = 2:1), Pt/CNTs-EG-DES (blue, ethylene glycol/choline chloride = 1:1), and Pt/CNTs-EG-DES (black, ethylene glycol/choline chloride = 1:2) in a 0.5 M H_2_SO_4_ + 0.5 M CH_3_OH solution. The Pt/CNTs-EG-DES (ethylene glycol/choline chloride = 2:1) has peak current densities (1101.2 mA mg^−1^_Pt_) that are higher than those on the Pt/CNTs-EG-DES (ethylene glycol/choline chloride = 1:1) (292.7 mA mg^−1^_Pt_) and Pt/CNTs-EG-DES (ethylene glycol/choline chloride = 2:1) (206.6 mA mg^−1^_Pt_) catalysts. The above results indicate that ethylene glycol/choline chloride = 2:1 is the optimal result, because the optimal ratio of DES formation is also the ratio of hydrogen bond donor to hydrogen bond acceptant, which is 2:1. As shown in Figure 4c, the peak current densities of methanol oxidation in the forward scan of the Pt/CNTs-EG-DES are also much higher than those on the Pt/CNTs-W (180.1 mA mg^−1^_Pt_) and Pt/C (82.6 mA mg^−1^_Pt_) catalysts. Expressly, the Pt/CNTs-EG-DES catalyst presents better MOR mass activity in comparison with the recent research works on Pt-based catalysts (Table S1). Figure 4d shows the current–time curves of the Pt/CNTs-EG-DES, Pt/CNTs-W, and Pt/C in a 0.5 M CH_3_OH + 0.5 M H_2_SO_4_ solution at 0.5 V for 3600 s. In all curves, the initial fast current decay indicates poisoning of the electrocatalysts, which is most likely due to the formation of intermediate species, such as CO_ads_, CH_3_OH_ads_, and CHO_ads_, during MOR [45]. After a long measurement period, the Pt/CNTs-EG-DES maintained a higher current density (365.2 mA mg^−1^_Pt_), which is approximately 2.0 and 2.97 times those of the Pt/CNT-W (150.1 mA mg^−1^_Pt_) and Pt/C (120.3 mA mg^−1^_Pt_) catalysts, respectively. After long-term testing, Figure S2 shows that Pt–O bonding is no longer obvious, indicating that Pt–O bonding participates in the reaction with the by-product (CO) during the durability process, which is also the reason why the Pt/CNT-EG-DES has better anti-toxicity. These results demonstrate that the Pt/CNTs-EG-DES catalyst exhibits higher electrocatalytic activity and long-term durability for MOR. The greatly enhanced electrocatalytic performance of the Pt/CNTs-EG-DES for the MOR may have been due to this special node structure that obtained more active sites and generated more Pt–O bonds, which changed the electronic states of the Pt atoms. The formation of oxygen-containing species (Pt–O bond) at a lower potential could transform CO-like poisoning species on Pt, releasing the active sites on Pt for a further electrochemical reaction, or the strong Pt–O interaction was related to the redox reaction between Pt and O, which enhanced the electrocatalytic performance of the Pt/CNTs-EG-DES for MOR. Figure S3 and Table S1 show the correlative ECSA, specific activity, mass activity, and onset of final activity in the stability tests for methanol electrooxidation.

**Figure 5:**
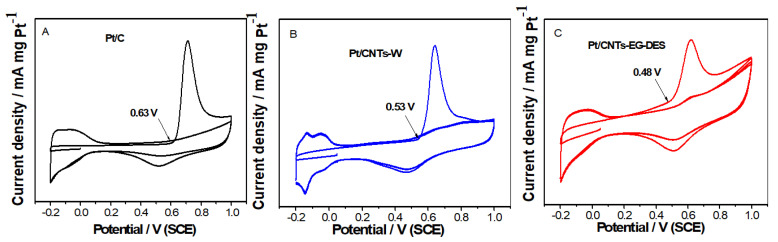
CO stripping voltammograms of Pt/C (**A**), Pt/CNTs-W (**B**), and Pt/CNTs-EG-DES (**C**) catalysts in 0.5 M H_2_SO_4_.

The CO tolerance of the as-prepared catalysts was also investigated using CO stripping experiments. Figure 5 shows the CO stripping voltammograms of the Pt/C (A), Pt/CNTs-W (B), and Pt/CNTs-EG-DES (C) in a 0.5 M H_2_SO_4_ solution. Prior to the oxidation of the adsorbed CO, the hydrogen adsorption/desorption was completely suppressed in all of the curves. However, the peaks associated with hydrogen adsorption/desorption appeared after the removal of the adsorbed CO. The onset potential of the adsorbed CO oxidation on the Pt/CNTs-EG-DES catalyst negatively shifted to 0.48 V, and the corresponding potentials were 0.53 V and 0.63 V on the Pt/CNT-W and Pt/C catalysts, respectively, indicating the superior CO tolerance of the Pt/CNTs-EG-DES.

The following are the details of how the EG-DES affects the long-term stability of the catalyst. Firstly, the viscosity environment of the DES has a special nodal structure, which is conducive to obtaining more active sites, enhancing the performance of the catalyst and maintaining good stability. Secondly, the special environment of the DES is conducive to obtaining more Pt–O bonds, which is conducive to the removal of CO and enhances the performance of the catalyst to maintain good stability. Thirdly, the DES contains abundant electroactive adsorption species such as choline cations, chloride ion, and urea, which preferentially adsorb on particular crystal faces through electrochemical adsorption, serving as a structure mediator in the shape-controlled synthesis of nanocrystals.

## 3. Experimental Section

### 3.1. Materials

Pristine MWCNTs with diameters of 40–60 nm, lengths of 5–15 mm, and purity of 98% were purchased from Shenzhen Nanotech Port Co., Ltd., (Shenzhen, China). A Nafion solution (5 wt%) was purchased from Sigma-Aldrich (St. Louis, MO, USA). Choline chloride [HOC_2_H_4_N(CH_3_)_3_Cl], ethylene glycol, absolute ethanol, H_2_PtCl_6_·6H_2_O, NaBH_4_, CH_3_OH, H_2_SO_4,_ and HNO_3_ were purchased from Shanghai Chemical Reagent Co., Ltd. (Shanghai, China), and all of the reagents used were analytically pure and used as received.

### 3.2. Preparation of Composite Catalysts

The Pt/CNTs-EG-DES catalyst was prepared according to the following method: The raw MWCNTs were treated using a well-known acid oxidation method (referred to as MWCNTs-AO) [39]. Appropriate H_2_PtCl_6_·6H_2_O and 20 mg acid-treated MWCNTs were ultrasonicated in 10 mL of EG-DES for 2 h. Then, excess NaBH_4_ was added to this suspension, and stirring was continued for an additional 5 h with the temperature at 60 °C. The solid products were separated from the mixture in a centrifuge and washed several times with absolute ethanol and tridistilled water until the filtrate was colorless. Then, these products were dried at 60 °C under vacuum overnight and transferred to the Pt/CNTs-EG-DES. By contrast, the Pt/CNTs-EG-DES-90 and Pt/CNTs-EG-DES-25 catalysts were prepared at 90 °C and 25 °C, respectively. For comparison, the Pt/CNTs-W was also prepared using a similar procedure in aqueous solutions. A commercial Pt/C was purchased from Sigma-Aldrich.

### 3.3. Physical Characterization

The X-ray diffraction (XRD) patterns were obtained using an X-ray diffractometer (Rigaku D/MAX 2500 v/pc, Rigaku Holdings, Akishima-shi, Japan) with a Cu Kα radiation source (λ = 1.5406 Å). The X-ray photoelectron spectroscopy (XPS) measurements were carried out using a Physical Electronics PHIQuantum 2000 system with an Al Kα radiation source, and all of the XPS spectra were calibrated with the C 1s line at 284.5 eV. The surface morphologies and microstructures of the prepared catalysts were analyzed using a high-resolution transmission electron microscope (HRTEM, JEOL JEM-2100) with an accelerating voltage of 200 kV. The Pt content analysis of different catalysts was performed using an inductively coupled plasma–optical emission spectrophotometer (ICP-OES, Thermo Electron IRIS Intrepid II XSP, Thermo Electron Corporation, Waltham, MA, USA). In this study, the Pt content (w%) of the as-prepared Pt/CNTs-EG-DES, Pt/CNT-W, and Pt/C catalysts was determined to be 18.6%, 17.1%, and 20.0%, respectively.

### 3.4. Electrochemical Measurements

**Scheme 1:**
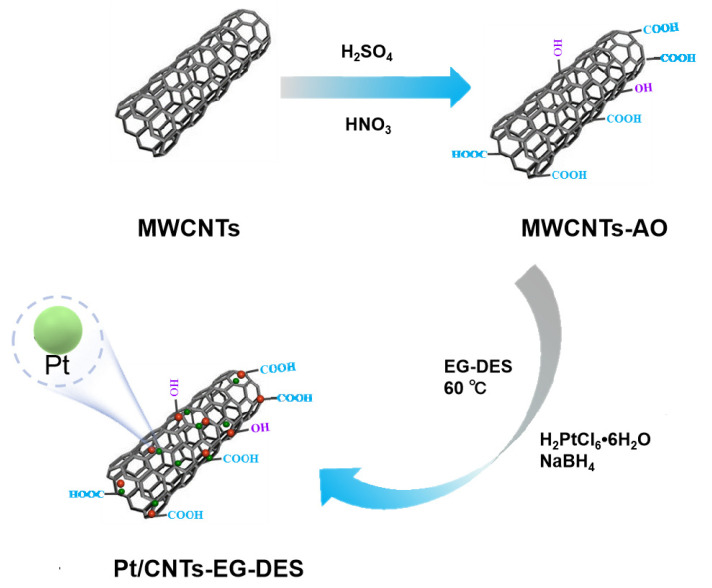
Schematic illustration showing the preparation of Pt/CNTs-EG-DES.

A glass carbon electrode modified with the obtained catalysts was prepared according to a previously published protocol [46]. The electrochemical measurements were performed at approximately 25 °C on a CHI 660D electrochemical workstation using a conventional three-electrode cell with platinum foil (1 cm^2^) and a saturated calomel electrode (SCE) as the counter and reference electrodes, respectively. All of the potentials in the electrochemical tests versus the SCE are reported. The electrocatalytic performance of the catalysts for MOR was studied in a 0.5 M CH_3_OH + 0.5 M H_2_SO_4_ solution. The CO stripping voltammograms were obtained by oxidizing preadsorbed CO in a 0.5 M H_2_SO_4_ solution at a scan rate of 50 mVs^−1^. Firstly, CO was bubbled into 0.5 M H_2_SO_4_ for 15 min to allow for the saturated adsorption of CO onto the catalyst while maintaining the potential scan between −0.2 and 0.0 V, and then the dissolved CO in the electrolyte was removed by purging with pure N_2_ for 20 min. In the electrochemical tests, the current density was expressed by the normalized current per milligram of Pt metal loading. Prior to each electrochemical experiment, the electrolytic solution was purged with pure N_2_ for 15 min, and a flux of N_2_ was maintained over the solution during measurements to prevent interference from atmospheric oxygen (Scheme 1).

## 4. Conclusions

Herein, a simple and green approach has been developed for the preparation of a Pt/CNTs-EG-DES. The green EG-DES plays an important role in generating many nodes and more Pt–O bonds, which make the Pt/CNTs-EG-DES exhibit an enhanced electrocatalytic performance (higher activity, increased long-term durability, and higher CO tolerance) compared to the Pt/CNT-W and Pt/C catalysts. This study demonstrates that the use of the EG-DES in favor of Pt nanoparticle nodes, and more Pt–O bonds, changed the ECSA of the catalyst, which is a good guide for fabricating a high-performance fuel cell catalyst.

## Data Availability

Data are available on request from the corresponding author.

## Supplementary Materials

The following supporting information can be downloaded at: https://www.mdpi.com/article/10.3390/molecules29215015/s1, Figure S1. TEM and HRTEM images of Pt/CNTs-W. Figure S2. Nodes of the Pt/CNTs-EG-DES catalyst. Figure S3. XPS survey spectra of the Pt/CNTs-W catalyst. Figure S4. Raman spectra of Pt/CNTs-EG-DES (red) and Pt/CNTs-W (black). Table S1. A recent literatures survey of the activity of MOR electrocatalysts [55,56,57,58,59,60,61,62,63].
