# Supplementary material for: Pt Nanoparticles on Multi-Walled Carbon Nanotubes with High CO Tolerance for Methanol Electrooxidation
Source: Molecules. 2024 Oct 23;29(21):5015. doi: 10.3390/molecules29215015 (PMC11547461; doi:10.3390/molecules29215015)
Supplement: Supplementary file 1 [file molecules-29-05015-s001.zip › molecules-3197805-supplementary.pdf]

Supporting information

Pt nanoparticles on multi-walled carbon nanotubes with High CO Tolerance for methanol electrooxidation

PingPing Yang[12], Shiming Dong[1], YouShu[1]*, Xuejiao Wei[3]*

[1]*College of Chemistry and Materials Engineering, Huaihua university, Huaihua, 418000, China*

[2]*College of Life Science and Chemistry, Hunan University of Technology, Zhuzhou, 412007, China*

[3] *Hutian middle school, Huaihua 418000, China*

*Corresponding author: * YouShu[1], E-mail: sy@hhtc.edu.cn;

*XueJiao Wei, E-mail: wei1348137@163.com

Table of Contents:

1. Supplementary Figures

Figure S1. TEM and HRTEM images of Pt/CNTs-W.

Figure S2. Nodes of the Pt/CNTs-EG-DES catalyst.

Figure S3. XPS survey spectra of the Pt/CNTs-W catalyst.

Figure S4 Raman spectra of Pt/CNTs-EG-DES (red) and Pt/CNTs-W (black).

Table S1 a recent literatures survey of the activity of MOR electrocatalysts.

2. Supplementary References

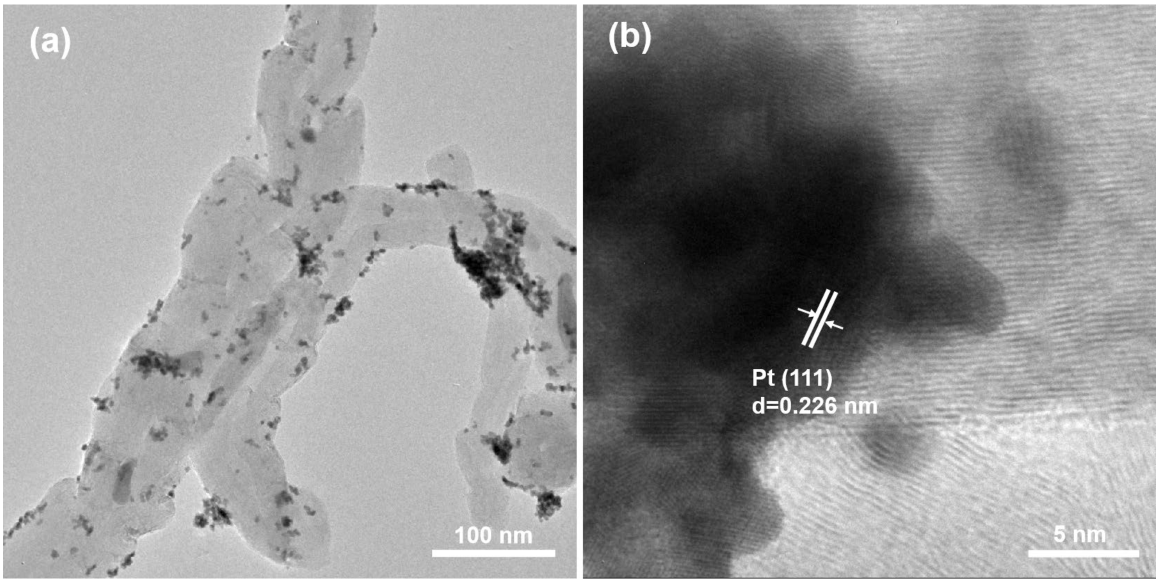

Figure S1. TEM and HRTEM images of Pt/CNTs-W catalyst.

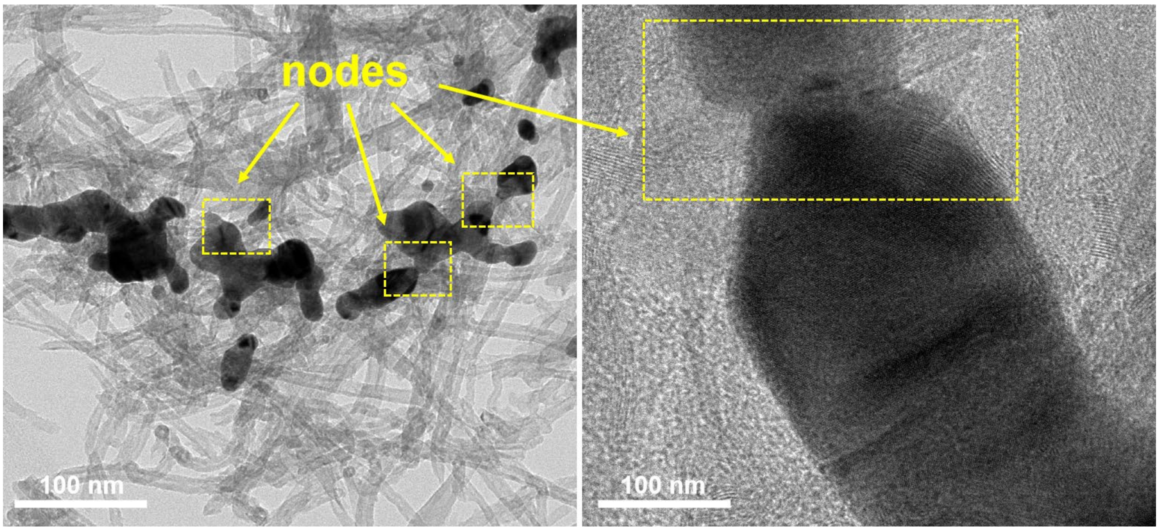

Figure S2. Nodes of the Pt/CNTs-EG-DES catalyst.

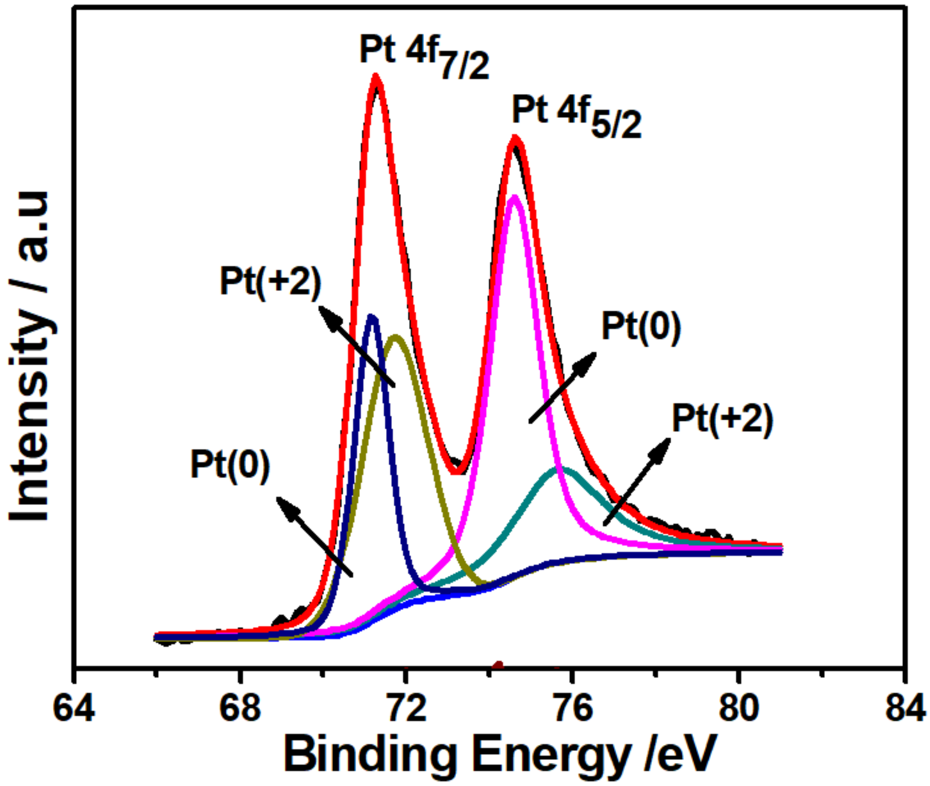

Figure S3. XPS survey spectra of the Pt/CNTs-W catalyst.

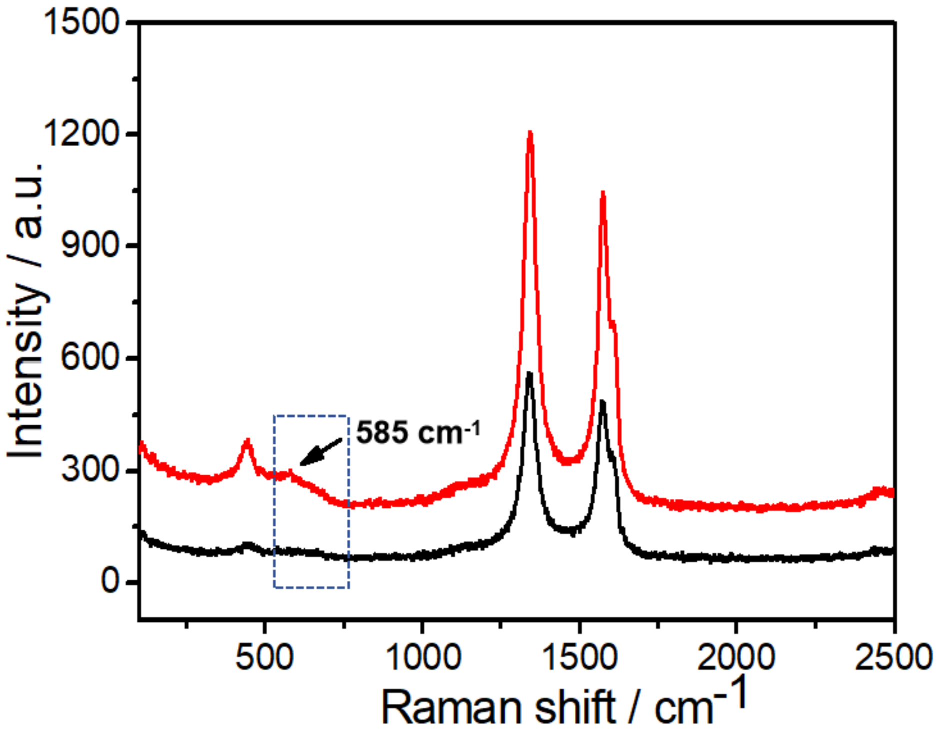

Figure S4 Raman spectra of Pt/CNTs-EG-DES (red) and Pt/CNTs-W (black).

Table S1 a recent literatures survey of the activity of MOR electrocatalysts.

Catalysts	Mass activity (mA mg^{-1} $_{Pt}$)	References
**Pt/CNTs-EG-DES**	**1101.2**	**This work**
Pt_3Sn_1-SnO_2/CNTs-D	361.2	[1]
Au@$Pt_{1.2}$Cu	180	[2]
PtRu/1-AP-MWCNTs	295	[3]
Pt Ru/APZ-MWCNTs	278.8	[4]
PtRu icosahedra	74.4	[5]
NP-$Pt_{70}Ru_{30}$	150	[6]
Commercial Pt/C	114.2	[7]
CB/PBI/PtRu	150	[8]
Pt/YBCPE	131	[9]
Pt–SnO_2(HS)	230.3	[10]

References

1. Yang, P, et al. One-step synthesis in deep eutectic solvents of Pt_3Sn_1-SnO_2 alloy nanopore on carbon nanotubes for boosting electro-catalytic methanol oxidation. *J Electroanal Chem* **2021,** 887, 115164.
2. Bian, T et al. Seed-mediated synthesis of Au@PtCu nanostars with rich twin defects as efficient and stable electrocatalysts for methanol oxidation reaction. *RSC Adv* **2019,** 9, (61), 35887-35894.
3. S Y Wang, et al. PtRu Nanoparticles Supported on 1-Aminopyrene-Functionalized Multiwalled Carbon Nanotubes and Their Electrocatalytic Activity for Methanol Oxidation. *Langmuir* 2008, 24, 18, 10505–10512.
4. Zhao, Y et al. Electrocatalytic oxidation of methanol at 2-aminophenoxazin-3-one-functionalized multiwalled carbon nanotubes supported PtRu nanoparticles. *Electrochim Acta* **2009,** 54, (27), 7114-7120.
5. Lin, Z et al. Facile synthesis of Ru-decorated Pt cubes and icosahedra as highly active electrocatalysts for methanol oxidation. *Nanoscale* **2016,** 8, (25), 12812-8.

6. Xu, C et al. Nanoporous PtRu alloys for electrocatalysis. *Langmuir* **2010,** 26, (10), 7437-43.
7. Guo, M et al. Hollow Pt skim-sandwiched Cu spheres supported on reduced graphene oxide-carbon nanotube architecture for efficient methanol electrooxidation. *Inter J Hydrogen Energ* **2019,** 44, (13), 6886-6895.
8. Zhao, L et al. Supramolecular assembly promoted synthesis of three-dimensional nitrogen doped graphene frameworks as efficient electrocatalyst for oxygen reduction reaction and methanol electrooxidation. *Appl Catal B: Environ* **2018,** 231, 224-233.
9. Zhang, J et al. PdPt bimetallic nanoparticles enabled by shape control with halide ions and their enhanced catalytic activities. *Nanoscale* **2016,** 8, (7), 3962-72.
10. Fan, Y et al. Hierarchical structure SnO_2 supported Pt nanoparticles as enhanced electrocatalyst for methanol oxidation. *Electrochim Acta* **2012,** 76, 475-479.
